# Small molecule RL71 targets SERCA2 at a novel site in the treatment of human colorectal cancer

**DOI:** 10.18632/oncotarget.6068

**Published:** 2015-10-10

**Authors:** Baofang Yang, Minxia Zhang, Jian Gao, Jiahuang Li, Lu Fan, Gang Xiang, Xingqi Wang, Xiaoning Wang, Xuefeng Wu, Yang Sun, Xudong Wu, Guang Liang, Yan Shen, Qiang Xu

**Affiliations:** ^1^ State Key Laboratory of Pharmaceutical Biotechnology, School of Life Sciences, Nanjing University, Nanjing, China; ^2^ Bioorganic and Medicinal Chemistry Research Center, School of Pharmaceutical Sciences, Wenzhou Medical College, Wenzhou, China

**Keywords:** RL71, SERCA2, novel binding site, colorectal cancer, targeted agent

## Abstract

While targeted agents are an important part of the treatment arsenal for colorectal cancer, there is still a lack of efficient small-molecule targeted agents based on the understanding of pathogenic molecular mechanisms. In this study, curcumin analog RL71 displayed potent cytotoxicity towards human colon cancer cells with an IC_50_ of 0.8 μM in SW480 cells and inhibited xenotransplanted tumor growth in a dose-dependent manner. Using affinity chromatography, we identified sarco/endoplasmic reticulum calcium-ATPase (SERCA) 2 as the binding target of RL71. Most notably, RL71 demonstrated special binding to SERCA2 at a novel site with the lowest estimated free energy −8.89 kcal mol^−1^, and the SERCA2 residues critical for RL71 binding were identified. RL71 suppressed the Ca^2+^-ATPase activity of SERCA2 both *in vitro* and *in vivo*, accompanied by the induction of endoplasmic reticulum stress leading to apoptosis and G2/M cycle arrest in SW480 cells. In addition, RL71 showed synergistic cytotoxicity with the pan-SERCA inhibitor thapsigargin. These results suggest that RL71 could be a selective small-molecule inhibitor of SERCA2, and that it may serve as a lead compound for the study of targeted colorectal cancer therapy.

## INTRODUCTION

Colorectal cancer (CRC) is one of the most important causes of cancer mortality [1]. Other than surgical resection, current therapy for CRC mainly relies on traditional cytotoxic agents with limited effects. These considerations highlight the importance of developing new treatments based on the understanding of pathogenic molecular mechanisms.

Endoplasmic reticulum (ER) calcium homeostasis is involved in a multitude of signaling pathways that control cell growth, differentiation and apoptosis [2]. Sarco/endoplasmic reticulum calcium-ATPases (SERCAs) act as Ca^2+^-ATPases that transfer Ca^2+^ from the cytosol to the lumen of the sarcoplasmic/endoplasmic reticulum at the expense of ATP hydrolysis. SERCA-dependent calcium transport is the only calcium-uptake mechanism in the ER, and therefore the regulation of SERCA activity is crucial to regulating calcium homeostasis in this organelle. Thus far, SERCA has been identified as a therapeutic target for prostate cancer and NOTCH1-mutated leukemia [3, 4]. Recent studies have reported that particular SERCA isoforms have altered expression patterns in various malignancies including CRC [5-7]. SERCA2 mRNA overexpression has been found both in cancerous tissues of CRC patients and in circulating tumor cells of relapsed CRC patients [8, 9]. Additionally, increased SERCA2 protein expression is significantly correlated with serosal invasion, lymph node metastasis, and advanced tumor stage. Consistently, our previous study also demonstrates that increased SERCA2 protein levels correlate strongly with tumor progression in patients with CRC [10]. Meanwhile, the loss of SERCA3 expression is an early event during the multistep process of colon carcinogenesis [11]. SERCA3 expression is low in colorectal carcinoma compared with that in the adjacent non-neoplastic mucosa and adenoma [12]. Thus, increased SERCA2 expression could be a rational and feasible target for anti-CRC drug development.

A variety of pan-SERCA inhibitors such as thapsigargin (TG) have been identified with diverse chemical structures and binding affinity [13]. Given the pervasive role of calcium signaling in normal physiology, only TG and related derivatives have been implemented in the clinic to date [14]. One strategy might be the development of isoform-specific small-molecule inhibitors of SERCA. The natural product curcumin has anti-CRC activity and is also a SERCA inhibitor that is 6-fold more effective in inhibiting SERCA2b than SERCA3a [15]. Therefore, investigating the selective potencies of curcumin analogs may prove to be useful.

In this study, we identified a potent anti-CRC compound RL71 (3,5-bis(3,4,5-trimethoxybenzylidene)-1-methylpiperidine-4-one) from a series of second-generation heterocyclic cyclohexanone curcumin analogs. Using affinity chromatography, we discovered that SERCA2 is the direct target of RL71. By computational virtual docking analysis and biological evaluation, RL71 was shown to interact with SERCA2 in the cleft on the lumenal side of the ER where Lys876 is critical for binding. The binding of RL71 at this novel site markedly inhibited SERCA2 activity and induced ER stress-associated apoptosis both *in vitro* and *in vivo*.

## RESULTS

### RL71 shows potent cytotoxicity towards human colon cancer cells

**Figure 1:**
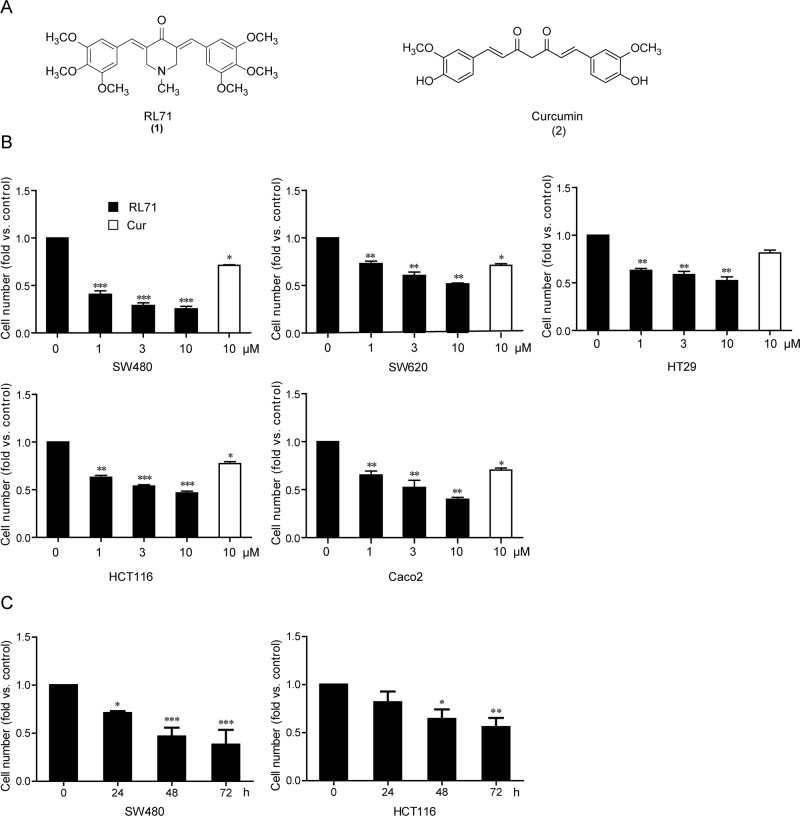
RL71 inhibits cell viability in human colon cancer cells **A.** Chemical structures of RL71 (1) and curcumin (2). **B.** Dose-dependent cytotoxicity of RL71 in human colon cancer cell lines. Cell viability was determined by MTT assay after a 48 h treatment. Curcumin (Cur) was used as a control. **C.** Time-dependent cytotoxicity of RL71 (1 μM) in SW480 and HCT116 cells. Data are the mean ± SEM of three independent experiments. *P < 0.05, **P < 0.01, ***P < 0.001 *versus* the control group without any treatment.

After a cell-based screen for cytotoxicity using a series of second-generation heterocyclic cyclohexanone curcumin analogs, we identified RL71 by its significant dose-dependent suppression of cell viability compared with curcumin in a panel of human colon cancer cell lines (Figure 1A, 1B; Table S1). Out of these cell lines, RL71 showed the lowest IC_50_ value (0.8 μM) for inhibition of cell viability in the colon carcinoma line SW480. Time-dependent inhibitory effects on cell viability were also confirmed in colon cancer cell lines SW480 and HCT116 (Figure 1C).

### Identification of SERCA2 as a cellular target of RL71

**Figure 2:**
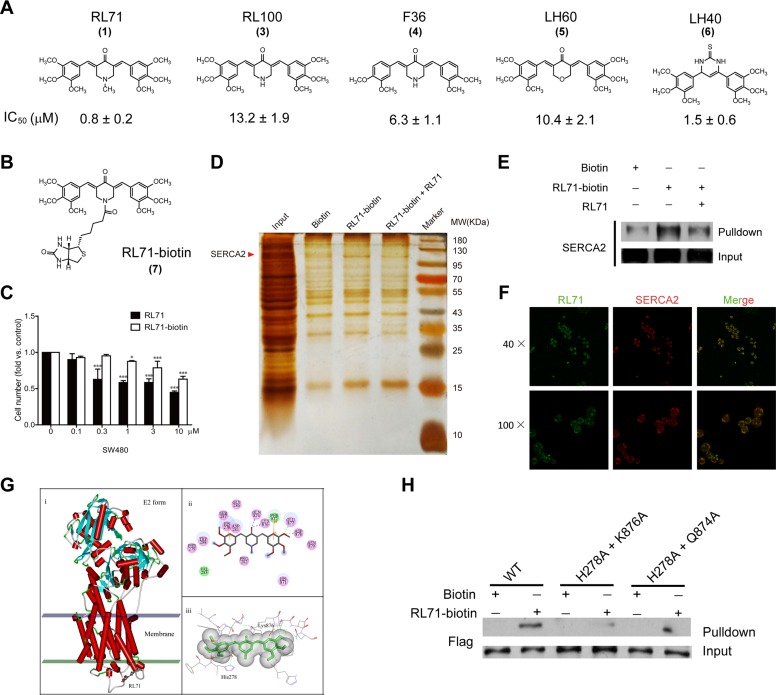
RL71 targets the SERCA2 protein **A.** Cytotoxicity of RL71 analogs (3-6) in SW480 cells. The concentrations that cause a 50% inhibition (IC_50_) of cell viability are shown. **B.** Chemical structure of biotinylated RL71 (RL71-biotin, 7). **C.** The effects of RL71 and RL71-biotin on SW480 cell viability. Cell viability was determined by MTT assay after a 24 h treatment. Data are the mean ± SEM of three independent experiments. *P < 0.05, ***P < 0.001 *versus* the control group without any treatment. **D.** Purification of RL71-binding proteins by affinity chromatography with 7-conjugated beads, which were incubated with the whole lysates of SW480 cells. Bound proteins were separated on an SDS gel and visualized by silver staining. **E.** Western blot analysis using the anti-SERCA2 antibody and the eluted proteins from **D.**. **F.** The co-localization of RL71 and SERCA2 in SW480 cells. The cells were treated with 10 μM of RL71 for 2 h and stained with an anti-SERCA2 antibody. Confocal microscopy was performed after a 2 h incubation. **G.** Molecular docking analysis of RL71 and SERCA2. **H.** The binding affinity for RL71-biotin of the double mutants within the binding site. The whole lysates of the HEK293 cells overexpressing FLAG-tagged SERCA2b (WT) or its mutants were incubated with RL71-biotin and streptavidin beads. The bound fractions were separated by SDS-PAGE and analyzed by western blotting.

To investigate the cellular target (or targets) of RL71, we attempted to generate biotinylated RL71 derivatives. Among 5 curcumin analogs, RL71 (1) exhibited the strongest cytotoxic effect on SW480 cells (Figure 2A). RL100 (3), which has a similar chemical structure except for the lack of a methyl substituent on the nitrogen, exhibited an approximate 16-fold weaker cytotoxicity than RL71. On the basis of their structures, we conjugated biotin to the nitrogen of RL71 to generate RL71-biotin (7) (Figure 2B). Although RL71-biotin showed decreased efficacy compared to RL71, RL71-biotin still maintained significant cytotoxic activity at 1 μM in SW480 cells (Figure 2C). Total cell extracts from SW480 cells were used for the affinity chromatograph experiment. The proteins between 95 and 130 kDa were specially purified on RL71-biotin-coupled streptavidin beads and competed with soluble RL71 (Figure 2D). LC/MS analysis revealed that SERCA2 is a binding protein of RL71. This finding was confirmed by western blot analysis using an anti-SERCA2 antibody (Figure 2E). The interaction between RL71-biotin and SERCA2 could be competed by RL71. Because of the innate fluorescent property of RL71, immunofluorescence staining was performed to identify its subcellular localization. We observed that RL71 co-localized with SERCA2 (Figure 2F). Additionally, the co-localization of RL71 and ER-tracker was revealed in SW480 cells (Figure S1). These results indicate that RL71 can potentially target SERCA2 in the ER.

To further explore the potential mode of binding of RL71 to the SERCA2 protein, we employed molecular docking using AutoDock 4.2. Based on the reported crystal structure of SERCA1 (Protein Data Bank (PDB) 3ar4) [16], we performed homology modeling for the SERCA2-RL71 complex, because the SERCA isoforms share over 75% homology at the amino acid level with similar domain structures. The model with the lowest estimated free energy for binding (−8.89 kcal mol^−1^) was selected. As shown in Figure 2G, RL71 appears to bind the cleft between the loop connecting M3 and M4 and the L78 loop on the lumenal side, possibly sealing the access pathway at the very surface through hydrogen bonds, hydrophilic interactions and Van der Waals forces with a group of residues. Our modeling analysis predicated that RL71 forms hydrogen bonds with Gln874, Lys876, Glu877 and Asp878. In addition, RL71's two phenyl rings could form Pi-Pi interactions with His278 and Phe872. To verify the importance of these residues in the binding of RL71, we generated SERCA2 mutants carrying H278A, Q874A and K876A mutations. As predicted by the model, the H278A and Q874A double mutant showed a reduced affinity for RL71-biotin (Figure 2H). Notably, we hardly detect any apparent binding between RL71-biotin and the H278A and K876A double mutant. These results indicate that RL71 possibly binds to SERCA2 at a novel site where amino acid Lys876 is critical for binding.

Our analysis also showed that the less active analog RL100 could dock into the same site in SERCA2 and form hydrogen bonds with side-chains of Gln874 and Lys876. It was observed that the nitrogen in RL100 could form a hydrogen bond with Asp281 (Figure S2). This interaction induces the deflection of the molecule, which broke hydrogen bonds with Glu877 and Asp878, and weakened the Pi-Pi interactions of His278 and Phe872.

### RL71 inhibits SERCA2 activity and induces ER stress-associated apoptosis

**Figure 3:**
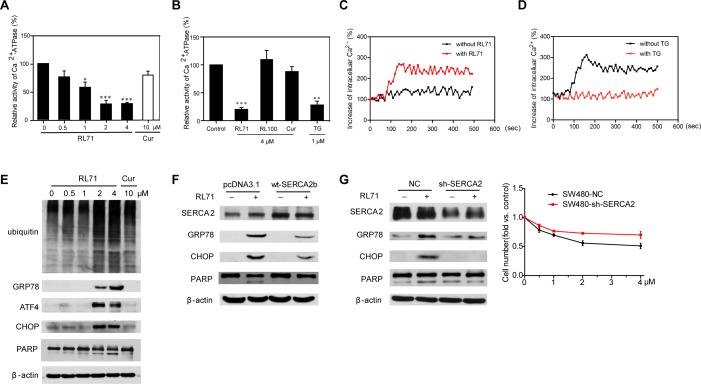
RL71 displays SERCA2-inhibiting activity and facilitates apoptosis by inducing ER stress in SW480 cells **A.** The dose-dependent inhibitory effect of RL71 on Ca^2+^-ATPase activity. The cells were incubated with the indicated concentrations of RL71 or Cur for 24 h. Then, the Ca^2+^-ATPase activity was measured according to the instructions of the Ca^2+^-ATPase kit provided by Nanjing Jiancheng Bioengineering Institute. **B.** The effects of the indicated compounds on Ca^2+^-ATPase activity. **C.** The increase of intracellular Ca^2+^ concentrations after RL71 treatment. Fura-2/AM loaded cells were stimulated with or without 2 μM of RL71. The y-axes represent the percentage of intracellular Ca^2+^ concentration. The x-axes depict the time in seconds, with time 0 representing the time of RL71 addition. The data are representative of at least 3 experiments. **D.** Changes of [Ca^2+^]_i_ after pretreatment with vehicle or with TG (5 μM), followed by stimulation with 2 μM of RL71. The data are representative of at least 3 experiments. **E.** The protein levels of ubiquitin-linked proteins, GRP78, ATF4, CHOP and PARP. Cells were treated with the indicated concentrations of RL71 or Cur for 24 h. **F.** The effects of SERCA2 overexpression on GRP78, CHOP and PARP expression. Cells were transiently transfected with pcDNA3.1 or SERCA2b expression plasmids. After 24 h, the cells were incubated with 2 μM of RL71 for another 24 h. **G.** The effects of SERCA2 knockdown on GRP78, CHOP and PARP expression (left panel) and cell viability (right panel). Cells stably expressing control virus or sh-SERCA2 virus were incubated with 2 μM of RL71 for 24 h. The protein levels were measured by western blot. The cell survival rate was determined by MTT assay. The data are mean ± SEM of three independent experiments. *P < 0.05, **P < 0.01, ***P < 0.001 *versus* control. All of the western blot analyses are representative of at least 3 experiments.

To test the effects of RL71 on SERCA2 function, we measured the Ca^2+^-ATPase activity in the treated SW480 cells. RL71 significantly inhibited the Ca^2+^-ATPase activity in a dose-dependent manner (Figure 3A). Additionally, 4 μM of RL71 inhibited the Ca^2+^-ATPase activity by 77%, to a level comparable to 1 μM of TG (Figure 3B). In contrast, the less active analog RL100 failed to show any inhibition. To further validate the inhibitory effect of RL71 on SERCA-associated Ca^2+^-uptake activity, we examined intracellular Ca^2+^ mobilization in treated SW480 cells. In Ca^2+^-free medium, RL71 induced a steady rise in cytosolic Ca^2+^ levels (Figure 3C). When the cells were pretreated with TG that depletes Ca^2+^ stores in ER, RL71-induced [Ca^2+^]_i_ increase was abolished (Figure 3D). These data suggest that the cytosolic influx is due to SERCA inhibition in ER, but not extracellular calcium influx.

The inhibition of SERCA causes a depletion of the ER calcium storage pool and a subsequent rise in cytosolic calcium levels, which results in ER stress leading to apoptosis [17]. We thus tested the effects of RL71 on the ER stress-associated apoptotic signaling pathway in SW480 cells. Hoechst staining demonstrated that apoptotic changes in morphology occurred, characterized by typical chromatin condensation and blebbing nuclei in RL71-treated SW480 cells (Figure S3A). RL71 treatment caused the accumulation of ubiquitinated proteins and increased GRP78, ATF4, and CHOP expression, as well as the cleavage of PARP, in a dose-dependent manner (Figure 3E). Because CHOP acts as a key transcription factor of DR5 under the condition of ER stress, we observed that RL71 treatment indeed caused an increase in DR5 protein levels (Figure S3B). The up-regulation of DR5, but not Fas, was confirmed on the surface of SW480 cells after RL71 treatment (Figure S3C). In addition, 4-phenyl-butyric acid (PBA), a chemical chaperone reported to be an inhibitor of ER stress [18], reduced CHOP expression in RL71-treated SW480 cells (Figure S3D).

To evaluate the involvement of SERCA2 in RL-71-induced ER stress, we examined the effects of overexpression and knockdown of SERCA2 on the expression of ER stress-related molecules in RL71-treated SW480 cells. SERCA2 (predominantly the ubiquitous SERCA2b isoform) is always present in cancer and leukemia cell lines [5, 11, 19]. Real time-PCR confirmed the predominant expression of SERCA2b, and RL71 showed no effect on SERCA2 expression at either mRNA or protein levels in SW480 cells (Figures S4A and S4B). The overexpression of SERCA2b reduced the elevation of GRP78, CHOP expression and the cleavage of PARP in RL71-treated SW480 cells (Figure 3F). Moreover, stable SERCA2 knockdown blocked their up-regulation (Figure 3G, left panel). RL71-associated cell growth inhibition was also reversed, at least in part, by SERCA2 knockdown (Figure 3G, right panel).

### RL71 targets SERCA2 at a novel site during ER stress-associated apoptosis

**Figure 4:**
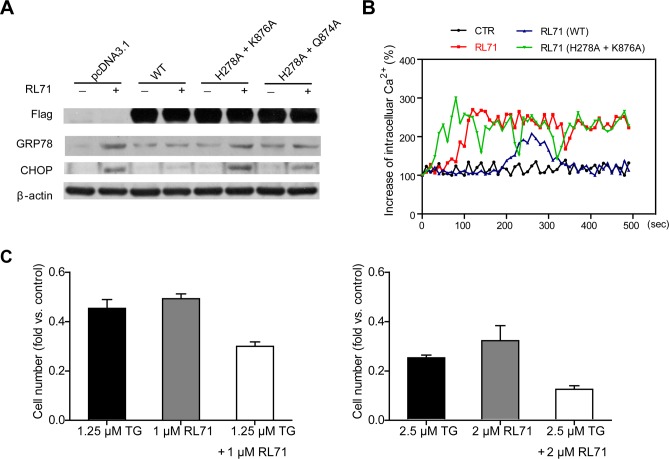
Binding of RL71 at a novel site contributes to ER stress-associated apoptosis **A.** The effects of SERCA2 mutants within the binding site on GRP78 and CHOP expression. The HEK293 cells were transiently transfected with FLAG-tagged SERCA2b or its mutant expression plasmids. After 24 h, the cells were incubated with 2 μM of RL71 for another 24 h. The data are representative of at least 3 experiments. **B.** The effects of the SERCA2 mutants on intracellular Ca^2+^ concentrations. Transfected HEK293 cells were loaded with Fura-2/AM and then stimulated with or without 2 μM of RL71. Increasing percentages of intracellular Ca^2+^ concentrations were monitored. The data are representative of at least 3 experiments. **C.** Synergistic cytotoxicity of RL71 with thapsigargin in SW480 cells. RL71 and thapsigargin (TG) were evaluated in a cell viability assay individually and in combination at the indicated concentrations. Cell viability was determined by MTT assay after a 48 h treatment. The data are the mean ± SEM of three independent experiments.

To clarify the mechanism of action of RL71 during the induction of ER stress, we investigated the effects of SERCA2 mutants on ER stress-related molecules in transfected HEK293 cells. Unlike wild type SERCA2b, the H278A and K876A double mutant failed to reduce the elevation of GRP78 and CHOP expression upon RL71 treatment. In contrast, the H278A and Q874A double mutant blocked the up-regulation of GRP78 and CHOP to a lesser extent than wild type SERCA2b (Figure 4A). In addition, the H278A and K876A double mutant did not decrease the cytosolic Ca^2+^ levels induced by RL71 (Figure 4B). These results were consistent with the binding affinities of the RL71-SERCA2 mutant interaction, indicating that RL71 interacts directly with SERCA2 in the cleft on the lumenal side of ER where Lys876 is critical for binding. The identity of this novel binding site was also supported by evidence showing that RL71 synergistic cytotoxicity with thapsigargin in SW480 cells (Figure 4C), which binds the E2 form of SERCA in the transmembrane domain [20].

### RL71 induces G2 cell-cycle arrest via ER stress

**Figure 5:**
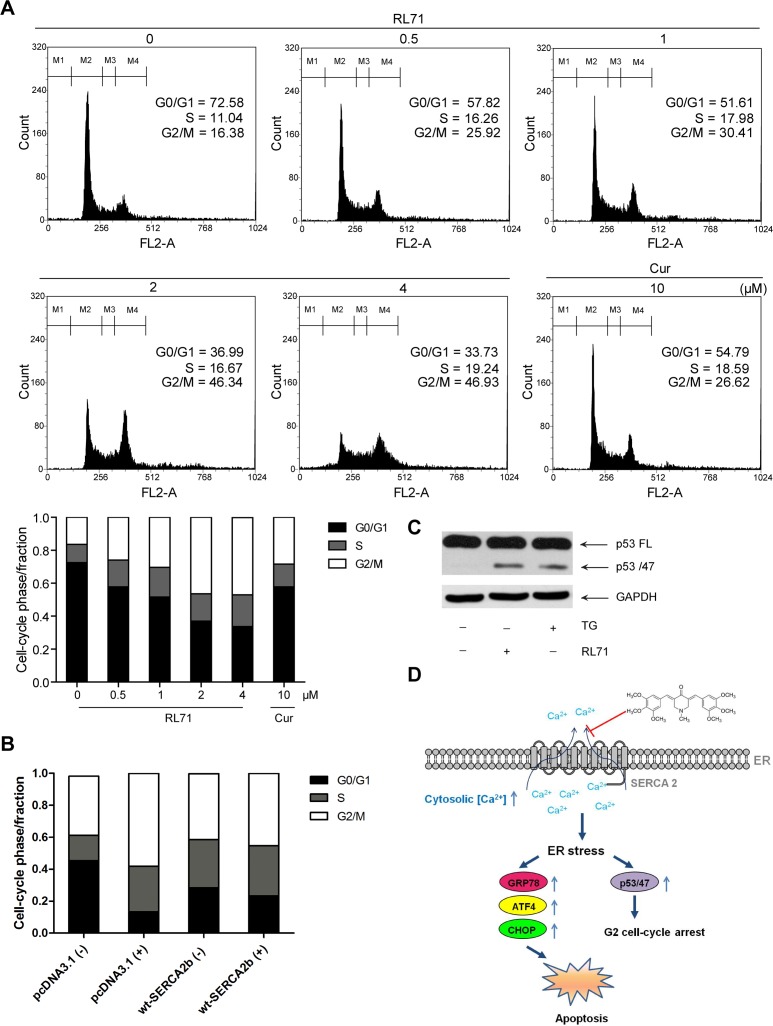
RL71 induces G2/M cell cycle arrest via ER stress in SW480 cells **A.** G2/M cell cycle arrest in RL71-treated SW480 cells. Cells were incubated with indicated concentrations of RL71 or Cur for 24 h. Propidium iodide staining and flow cytometry were used to determine the proportion of cells in various phases of the cell cycle. **B.** The effects of SERCA2 overexpression on cell cycle arrest. SW480 cells were transiently transfected with pcDNA3.1 or SERCA2b expression plasmids. After 24 h, the cells were incubated with 2 μM of RL71 for another 24 h. **C.** The protein levels of the full-length p53 (p53FL) and the p53 isoform p53/47. Cells were incubated with 2 μM of RL71 or 1 μM of TG for 24 h. The data are representative of at least 3 experiments. **D.** Schematic representation of the mechanism by which RL71 affects ER stress-associated signaling events by targeting SERCA2.

Treating cells with drugs that target ER calcium influx results in ER stress, which induces G2 cell-cycle arrest via mRNA translation of the p53 isoform p53/47 [21]. Measurement of DNA content showed that RL71 indeed increased the percentage of cells in G2/M phase and decreased the percentage in G0/G1 phase of the cell cycle in a dose-dependent manner (Figure 5A). An average increase of 60% in the proportion of SW480 cells in G2/M phase over control was observed upon incubation with RL71 (0.5 μM) or curcumin (10 μM). The overexpression of SERCA2b suppressed G2 cell-cycle arrest in RL71-treated SW480 cells (Figure 5B). Like TG, RL71 induced p53/p47 mRNA translation (Figure 5C). These results demonstrate that RL71 induces ER stress by interfering with SERCA2 function (Figure 5D).

### RL71 suppresses SW480 cell growth *in vivo* via inhibition of SERCA2 activity

**Figure 6:**
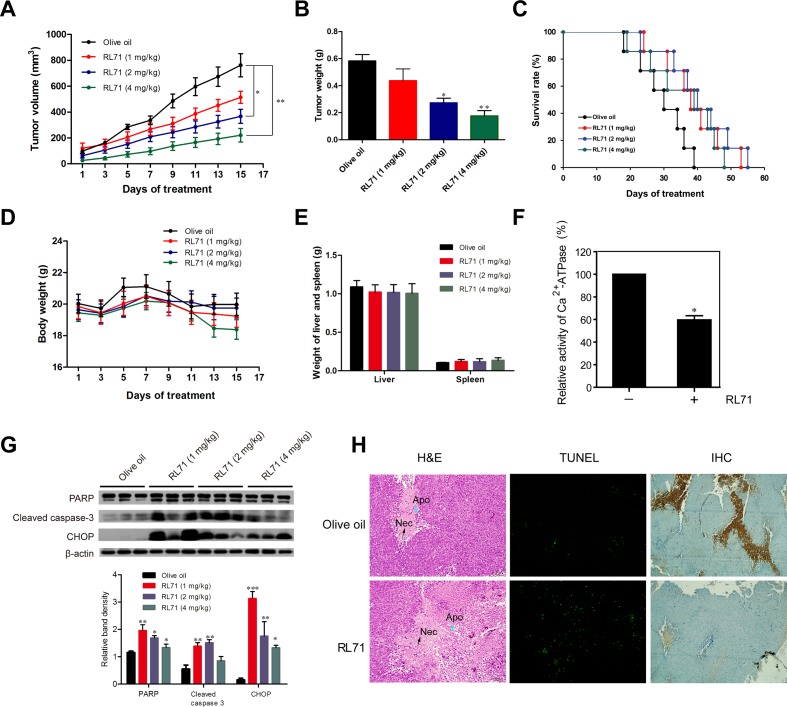
RL71 suppresses the growth of SW480 cells in nude mice via inhibition of SERCA2 activity **A.** Tumor volumes. SW480 cells were injected subcutaneously into the right flank of nude mice. Two weeks later, the tumor-bearing mice were distributed into 4 groups and treated with various doses of RL71 or olive oil for 14 additional days. The tumor volumes were monitored and recorded every two days (*n* = 8-10 mice per group). **B.** Tumor weight on day 15 of treatment. **C.** Survival curve of mice (*n* = 14 mice per group). **D.** Body weight. **E.** Weight of liver and spleen on day 15. **F.** The Ca^2+^-ATPase activity of tumor samples from RL71-treated mice at 2 mg/kg and olive oil-treated controls. The tumor tissues were excised on day 15 and measured according to the instructions of the Ca^2+^-ATPase kit. **G.** Protein levels of PARP, cleaved caspase-3 and CHOP in tumor samples. The tumor tissues were excised on day 15 and analyzed by western blot. **H.** Tumor tissues stained with H&E (left), TUNEL reagents (middle) or an antibody specific for CD31 (right). The tumor tissues were excised on day 15. The data shown are representative of three experiments. Necrotic tumor cells (Nec) and apoptotic condensed nuclei (Apo) are indicated with arrows. Original magnification, ×100. The data are the mean ± SEM of 8-10 mice per group. *P < 0.05, **P < 0.01, ***P < 0.001 *versus* the olive oil group.

To test RL71 activity *in vivo*, we subcutaneously inoculated SW480 cells into the right flank of nude mice. Two weeks later, when the tumors began to grow (approximately 50 mm^3^), the mice were randomized into 4 groups, olive oil control and RL71 (1, 2 and 4 mg/kg). As shown in Figure 6A, the administration of RL71 inhibited tumor growth compared with controls in a dose-dependent manner. When the tumors were removed on day 15, the average weight of tumors from RL71-treated mice at the 4 mg/kg dose was 3-fold less than the olive oil-treated tumors (Figure 6B). The overall survival of the tumor-bearing mice with RL71 treatment was prolonged than controls (Figure 6C). RL71 did not have significant effect on the body weight (Figure 6D), as well as the weight of the liver or spleen (Figure 6E). In addition, Ca^2+^-ATPase activity was inhibited by about 40% in the RL71-treated tumors at 2 mg/kg compared with the olive oil-treated tumors (Figure 6F), confirming SERCA2 inhibition *in vivo*. The cleavage of PARP and caspase 3, as well as CHOP expression in the tumors, were greatly elevated in the RL71 treatment groups (Figure 6G). Moreover, the histochemical and TUNEL staining assay showed apparent apoptotic induction in tumor tissues in the RL71-treated group at 2 mg/kg compared with the olive oil-treated group (Figure 6H). Immunostaining also revealed that CD31^+^-marked blood vessels in RL71-treated mice were greatly decreased compared with controls.

## DISCUSSION

While targeted agents are an important part of the treatment arsenal for CRC, few small-molecule agents that target intracellular signaling pathways have been shown to improve the outcome of patients, particularly with metastatic CRC [22]. It has been postulated that using predictive biomarkers for CRC could be indispensable for determining which patients will benefit from these target-based therapies. Our study and other recent work demonstrate that increased levels of SERCA2 expression may be a tumor marker during CRC progression [8-10]. In the present study, we report the discovery of the small molecule RL71 as a potent anti-CRC compound and identified SERCA2 as the direct target of RL71.

With a combined approach that included affinity chromatography, biochemical analysis and structural modeling, we show that RL71 binds to SERCA2 at the cleft between the loop connecting M3 and M4 and the L78 loop on the lumenal side of the ER (Figure 2). Classic pan-SERCA inhibitors thapsigargin and cyclopiazonic acid are both reported to bind in the transmembrane domain, and each induces unique changes to the cytoplasmic headpiece [23]. Another SERCA inhibitor, 2APB, can bind close to the cytoplasmic loop between M6 and M7 (L67) and possibly block Ca^2+^ from entering Ca^2+^-binding sites [24]. It is likely that when RL71 binds to this novel site, it acts as a ‘plug,’ sealing the access pathway/route by which Ca^2+^ can enter the ER. Mutation of the residues in the binding site revealed that the amino acid Lys876 in the L78 loop was the most critical for the specific binding of RL71 and also for the activities of RL71 acting on ER stress-related signaling and the cytosolic Ca^2+^ levels (Figures 2 and 4). Comparison of protein sequences of the L78 loop among the human SERCA 1-3 isoforms showed that Lys876 is only located in SERCA2 (data not shown). Because paired inhibitor studies have demonstrated that a change in SERCA conformation upon binding of a single inhibitor can favor the binding of a second inhibitor [25], a uniquely synergistic relationship was uncovered between RL71 and thapsigargin in cytotoxicity, further confirming a novel binding site for RL71. In addition, RL71 showed no inhibitory effect on the mRNA levels of SERCA3 and PMCAs that are located on the Golgi membrane and also involved in cellular Ca^2+^ homoeostasis (Figures S4C and S4D). Pre-inhibition of SERCA activity with TG completely abolished RL71-induced [Ca^2+^]_i_ increase (Figure 3D). Taken together, these results suggest that RL71 could be a novel SERCA2-specific inhibitor.

In this study, we found that RL71 induces apoptosis and promotes G2/M cell cycle arrest in SW480 cells (Figures 3 and 5). These results could be due to the induction of ER stress by RL71 via inhibition of SERCA2. With the exception of the unfolded protein response, imbalance of Ca^2+^ transfer also induces ER stress [26]. SERCA inhibition causes prolonged elevation of cytosolic calcium that elicits an ER stress response typified by the accumulation of unfolded proteins, CHOP induction and caspase activation [17]. Indeed, a steady rise in cytosolic Ca^2+^ levels was detected in RL71-treated SW480 cells. Meanwhile, the accumulation of ubiquitinated proteins and an elevation of GRP78, ATF4 and CHOP were detected. SERCA2 overexpression or knockdown impaired the induction of GRP78 and CHOP as well as the cleavage of PARP by RL71. It has been recently demonstrated that p53 can interact with SERCA pump at the ER, thus stimulating Ca^2+^ accumulation in the ER and allowing for apoptosis [27, 28]. However, RL71 inhibited SERCA2 activity and induced p53/p47 mRNA translation, suggesting that RL71 might induce p53/47-dependent G2 cell-cycle arrest via ER stress. Consistent with our results, a recent study showed that RL71 induced G2/M cell cycle arrest and apoptosis in breast cancer cells [29].

An *in vivo* test demonstrated that RL71 treatment dose-dependently inhibited tumor growth and reduced tumor weight in a SW480 xenograft model, but had little effect on the body weight as well as the liver or spleen weight of tumor-bearing mice (Figure 6). These results indicate that RL71 has relatively low toxicity in animals. In fact, a previous study showed that RL71 was orally available following a single oral dose of 8.5 mg/kg [29]. In line with the *in vitro* results, the inhibition of Ca^2+^-ATPase activity and the elevation of CHOP expression and apoptotic induction were detected in the RL71-treated tumor tissues. Moreover, CD31^+^ blood vessels were greatly decreased, indicative of an anti-angiogenic microenvironment. This finding is supported by evidence that RL71 inhibits HUVEC cell migration and the ability of these cells to form tube-like networks [29]. We also found the inhibitory effect of RL71 on cell migration in SW480 cells using wound healing assay (Figure S5).

Although SERCA inhibition has been postulated to provide a potential targeting strategy for anti-cancer therapy, most small molecule SERCA inhibitors are non-selective for SERCA isoforms, which prevents their immediate clinical application. For example, TG shows toxicity in normal cells in response to SERCA inhibition [6]. Only when it was used as a prodrug by conjugation with peptides unique to prostate-specific antigen enzyme, successful targeted therapy of prostate cancer was achieved in clinical trial [3]. Curcumin is a potent SERCA2 inhibitor, but has shown limited clinical efficacy due to its low bioavailability and low stability in physiological media [19, 30, 31]. The successful synthesis of second generation heterocyclic cyclohexanone curcumin analogs affords good candidates, since they have enhanced activity and stability in biological medium compared with curcumin [32, 33]. In this study, RL71 showed potent anti-CRC activity both *in vitro* and *in vivo* over other curcumin analogs, which is possibly related to the binding affinities for SERCA2. Furthermore, RL71 also repressed the growth of other human cancer cells (Figure S6). These results implicate its clinical therapy potential.

In summary, this study demonstrates that curcumin analog RL71 interacts with SERCA2 at a novel binding site. Thus binding might contribute to the selective potency on SERCA2 and impaired toxicity of RL71. The study also demonstrates the efficacy of SERCA2 as a therapeutic target for the treatment of CRC and suggests that RL71 may serve as a tool to study isoform-specific SERCA inhibition.

## MATERIALS AND METHODS

### Reagents

RL71, RL100 (3,5-bis(3,4,5-trimethoxybenzylidene)- piperidine-4-one), F36 (3,5-bis(3,4-dimethoxybenzylidene)- piperidine-4-one), LH60 (3, 5- di(3, 4, 5- trimethoxybenzylidene) tetrahydro- 2H- pyran- 4- one), LH40 (3, 4- dihydro- 4, 6- bis(3, 4, 5- trimethoxyphenyl) - 2(1H) - pyrimidinethione) and other synthetic curcumin analogs were kindly provided by Professor Guang Liang from Wenzhou Medical College, China. These structures were confirmed by comparing MS, ^1^H NMR and physical data with those reported in the literature [32, 34]. The purity is higher than 97%. Curcumin (>98% purity), 5-Diphenyl-2H-tetrazolium bromide (MTT) and thapsigargin were purchased from Sigma-Aldrich (St. Louis, MO). ER-tracker Red and Fura-2/AM were purchased from Beyotime (Nanjing).

### Cell culture

Human colon carcinoma cell lines SW480, SW620, HT29, HCT116 and Caco2 were purchased from the American Type Culture Collection. HEK293 cells were purchased from the Shanghai Institute of Cell Biology (Shanghai, China). The cell lines were maintained in DMEM supplemented with 10% fetal bovine serum (FBS, Life Technologies), 100 U/mL penicillin, and 100 mg/mL streptomycin and incubated at 37°C in a humidified atmosphere containing 5% CO_2_.

### Mice

Eight-week-old NCR-nu / nu (nude) female mice were purchased from the Shanghai Laboratory Animal Center. Animal care was performed in compliance with the guidelines of the Ministry of Science and Technology of China (2006) and the related ethical regulations of Nanjing University. All efforts were made to minimize animal suffering and the number of animals used.

### Synthesis of 7

To identify RL71-interacting proteins using affinity chromatography, RL71 conjugated with a biotin molecule was chemically synthesized. RL100 (1 mmol), biotin (1.2 mmol), TEA (1.5 mmol), EDCI (1.2 mmol), HOBt (1.2 mmol) and DCM (20 mL) were added to an RBF. The reaction was stirred at r.t. for 16 h. The reaction was quenched by sq. NH_4_Cl (20 mL) and extracted with ethyl acetate (15 mL × 3). The organic phase was dried by Na_2_SO_4_, concentrated, then purified by flash chromatography (petroleum ether/AcOEt: 3/1 v/v) to give the desired conjugate 6 (72%) as a light yellow solid. ^1^H NMR (400 MHz, CDCl_3_) δ: 7.77 (d, J = 21.9 Hz, 2H), 6.67 (d, J = 46.7 Hz, 4H), 5.63 (s, 1H), 5.11 (s, 1H), 4.93 (s, 2H), 4.74 (s, 2H), 4.45 (m, 1H), 4.27 (m, 1H), 3.91 (m, 19H), 3.05 (m, 1H), 2.86 (dd, J = 12.7, 4.8 Hz, 1H), 2.68 (m, 1H), 2.22 (t, J = 7.2 Hz, 2H), 1.52 (m, 4H), 1.27 (m, 3H). ESI-MS m/z: 682 [M + H]^+^.

### MTT assay

Cells were incubated with various concentrations of RL71 or curcumin at a density of 2×10^4^ cells per well in 96-well plates. In total, 20 μl of MTT (4 mg/mL in PBS) was added per well 4 h before the end of the incubation. MTT formazan production was dissolved by DMSO replacing the medium. The optical density at 570 nm was measured using a FL × 800 Fluorescence Microplate Reader (BioTek, Winooski, VT).

### Target identification of RL71 using affinity chromatography

As described previously [35], SW480 whole-cell lysates were incubated with 10 μM biotin, 10 μM RL71-biotin or 10 μM RL71-biotin plus 200 μM RL71 and 20 μl streptavidin-conjugated sepharose beads (GE Healthcare) at 4°C overnight for 12 h. After an extensive wash with PBS, the beads were boiled in 2× loading buffer (100 mM Tris-HCl (pH 6.8), 4% SDS, 1% bromophenol blue, 20% glycerol and 2% β-mercaptoethanol). Then, the supernatants were collected and separated with SDS-PAGE prior to silver staining. The band between 95 and 130 kD specific to RL71-biotin was excised from the gel and analyzed with LC/MS, which was performed by the Shanghai Institute of Biochemistry Proteomics Center (Shanghai).

### Western blot

Western blot analysis was performed as previously described [19]. Antibodies to SERCA2 (#9580), PARP (#9542), cleaved caspase-3 (#9664), DR5 (#3696), CHOP (#2895), p53 (#2524), ubiquitin (# 3933), and FLag (# 2368) were purchased from Cell Signal Technology (Beverly, MA), and antibodies to GRP78 (sc-13968), ATF4 (sc-22800), β-actin (sc-47778) and GAPDH (sc-166574) were from Santa Cruz Biotechnology (Santa Cruz, CA). Final detection was performed using the LumiGLO chemiluminescent substrate system (KPL, Guildford, UK).

### Confocal microscopy

SW480 cells were incubated with 10 μM of RL71 for 2 h and then fixed in 4% paraformaldehyde (pH 7.4) for 10 min at 37°C. Subcelluar localization of RL71 was analyzed following the manufacturer's protocol. For analysis of the co-localization of RL71 and SERCA2, the fixed cells were stained with an anti-SERCA2 antibody and detected with a secondary antibody. The fluorescent signals were detected using a FluoViewTM FV1000 confocal microscope (Olympus Corporation, Shinjuku, Tokyo, Japan) and analyzed by the Olympus Fluview Ver1.7b viewer (Olympus Corporation, Shinjuku, Tokyo).

### Construction of SERCA2b and SERCA2b mutants

The full length of human SERCA2b was subcloned into pcDNA3.1(+) as previously described in detail [19]. The amphimutation plasmids, pcDNA3.1(+)-mSERCA2b (His278→Ala; Lys876→Ala) and pcDNA3.1(+)-mSERCA2b (His278→Ala; GLN874→Ala), were constructed by PCR-based mutagenesis using the appropriate synthetic oligonucleotides and human SERCA2b cDNA as a template. The resulting FLAG-labeled fragments were inserted into the pcDNA3.1(+) vector. All of the mutations were confirmed by sequencing.

### *In vitro* binding assay

The HEK293 cells overexpressing FLAG-tagged SERCA2b or its mutants were lysed in protein lysis buffer (50 mM Tris-HCl, pH 7.4, 150 mM NaCl, 1% (v/v) Triton X-100, 5 mM EDTA and proteinase inhibitors) and centrifuged at 16,000 g for 15 min at 4°C. The supernatant was incubated with biotin or RL71-biotin and streptavidin beads. After an extensive wash with PBS, the bound fractions were separated by SDS-PAGE and analyzed by western blotting.

### Intracellular Ca^2+^ measurement

[Ca^2+^]_i_ was determined using the Ca^2+^-sensitive fluorescent indicator Fura-2/AM as previously described [36]. Briefly, cells were washed with HEPES-buffered medium (140 mM NaCl, 5 mM KCl, 1 mM Na_2_HPO_4_, 1 mM MgCl_2_, 1 mg/mL glucose and 20 mM HEPES, pH 7.4) and incubated with 2.5 μM Fura-2/AM for 30 min at 37°C. Then, the cells were washed twice and incubated with RL71 for another 15 min. Fluorescence intensity was monitored at 510 nm (5 nm slit) with alternated excitation at 340 nm and 380 nm (5 nm slit) using a TECAN GENIOS (Model Safire; Tecan, Switzerland). [Ca^2+^]_i_ was calculated using the formula: Intracellular free Ca^2+^ concentration = Kd (F0 / Fs) (R - R_min_) / (R_max_ - R). Kd represents a dissociation constant with a value of 224 nM. F0 and Fs represent the fluorescence intensity measured when the Ca^2+^ is at zero and at the saturation point, respectively. R is the observed fluorescence ratio. R_max_ and R_min_ are the maximum and minimum fluorescence ratios, respectively. Maximal and minimal fluorescence values were obtained at the end of the experiment by sequential addition of 0.1% Triton X-100 and 5 mM EGTA. In some experiment, Fura-2 loaded cells were pretreated with 5 μM TG for 10 min, followed by stimulation with RL71 in Ca^2+^-free medium. Changes in [Ca^2+^]_I_ were monitored.

### Lentivirus-mediated short hairpin RNA knockdown

Lentiviral vectors HPC003cpLKD.CMV.G&PR.U6.NC without (NC) or with a short hairpin RNA targeting SERCA2 (sh-SERCA2) were purchased from Neuronbiotech (Shang Hai). The sequence of SERCA2 shRNA is as follows: 5′-CAAAGUUCCUGCUGAUAUA-3′. SW480 cells were infected with the viral vectors and then maintained in puromycin at a concentration of 8 μg/mL. Resistant cells were allowed to grow for 2 weeks. The cells stably expressing the shRNA were selected.

### Analysis of the cell cycle

The cell cycle was analyzed by measuring propidium iodide (PI) staining using a FACSCalibur flow cytometer (Becton Dickinson, San Jose, CA). DNA contents of stained cells were analyzed with ModFit software (Becton Dickinson).

### Transplantation of SW480 cells into nude mice

Cultured SW480 cells were washed with and resuspended in ice-cold PBS. Portions of the suspension (3 × 10^6^ cells in 0.1 mL) were injected into the right flank of nude mice. Two weeks after the injection, the mice bearing tumors (an average size of 50 mm^3^) were distributed into 4 groups (*n* = 8-10 mice per group). RL71, dissolved in olive oil, was administered daily for 14 days by intraperitoneal injection at a dose of 1, 2 or 4 mg/kg. Tumor volumes were measured every 2 days and calculated using the following formula: 0.5236 × L1 × (L2)^2^, where L1 and L2 are the long and short diameters of the tumor mass, respectively. Tumor tissues, liver and spleen were excised and weighted on day 15. In another experiment, survival tests were made (*n* = 14 mice per group) as above and monitored daily until all the mice died.

### Immunohistochemistry and TUNEL assay

Immunostaining of CD31 was performed using a Real Envision Detection kit from the Gene Tech Company according to the manufacturer's instructions. H&E staining in tumor tissues was performed following the manufacturer's protocol. A TUNEL assay was performed to detect apoptotic cells using the TUNEL BrightGreen Apoptosis Detection kit from Vazyme (Piscataway, NJ) according to the manufacturer's instructions.

### Statistical analysis

The data are the mean ± SEM of three independent experiments. In some experiments, statistical analyses were performed using one-way analysis of variance (ANOVA) followed by a post-hoc test. Two-way analysis of ANOVA was used in the experiments containing 2 factors. The Kaplan-Meier method was used to evaluate the survival rate. *P* < 0.05 was considered significant.

## SUPPLEMENTARY FIGURES AND TABLES


