# Supplementary material for: Small molecule RL71 targets SERCA2 at a novel site in the treatment of human colorectal cancer
Source: Oncotarget. 2015 Oct 10;6(35):37613–25. doi: 10.18632/oncotarget.6068 (PMC4741952; doi:10.18632/oncotarget.6068)
Supplement: Supplementary file 1 [file oncotarget-06-37613-s001.pdf]

Small molecule RL71 targets SERCA2 at a novel site in the treatment of human colorectal cancer

Supplementary Material

Supplementary Figure S1

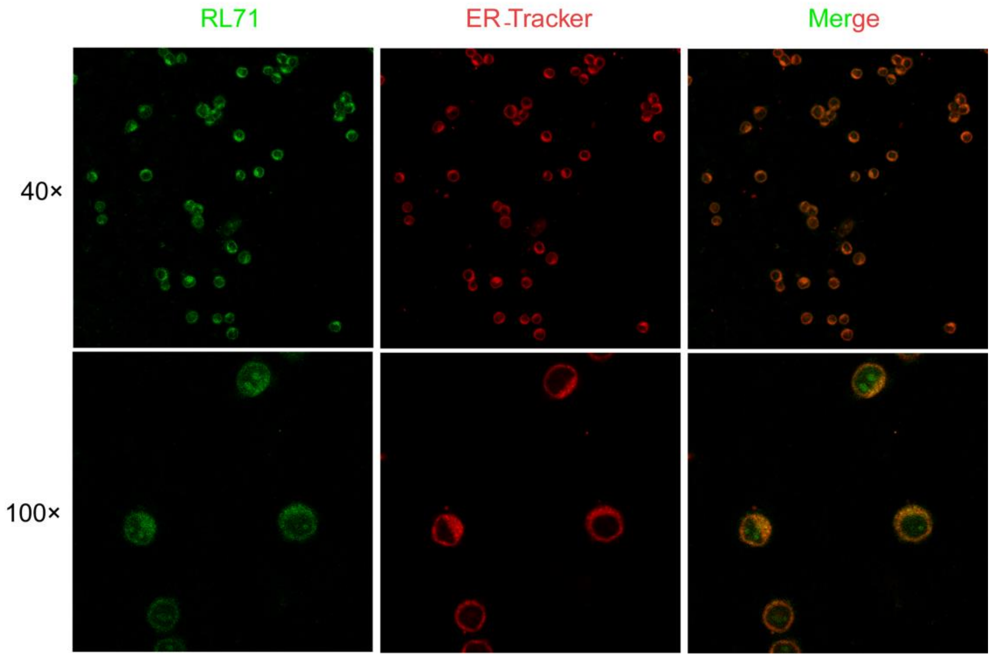

Figure S1 RL71 is located on the endoplasmic reticulum in SW480 cells. Cells were treated with 10 μM RL71 for 2 h and stained with ER-tracker to show the location of the endoplasmic reticulum. Confocal microscopy was performed after a 2 h incubation.

Supplementary Figure S2

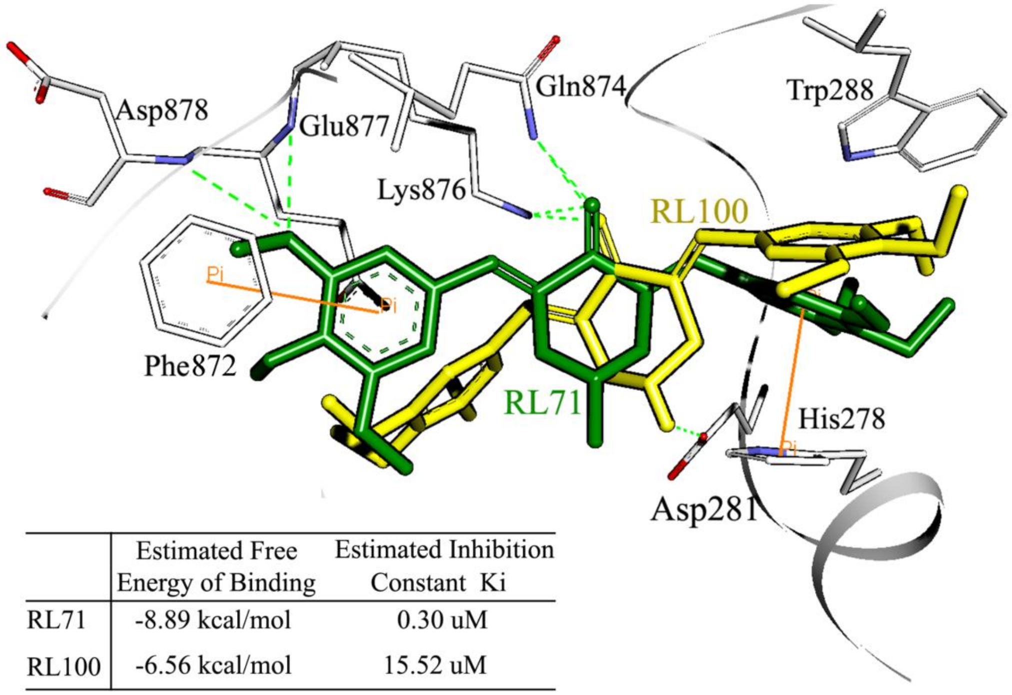

	Estimated Free Energy of Binding	Estimated Inhibition Constant Ki
RL71	-8.89 kcal/mol	0.30 uM
RL100	-6.56 kcal/mol	15.52 uM

Figure S2 Comparison of the binding activity between RL71 and RL100 to SERCA2. Molecular docking analysis was performed.

Supplementary Figure S3

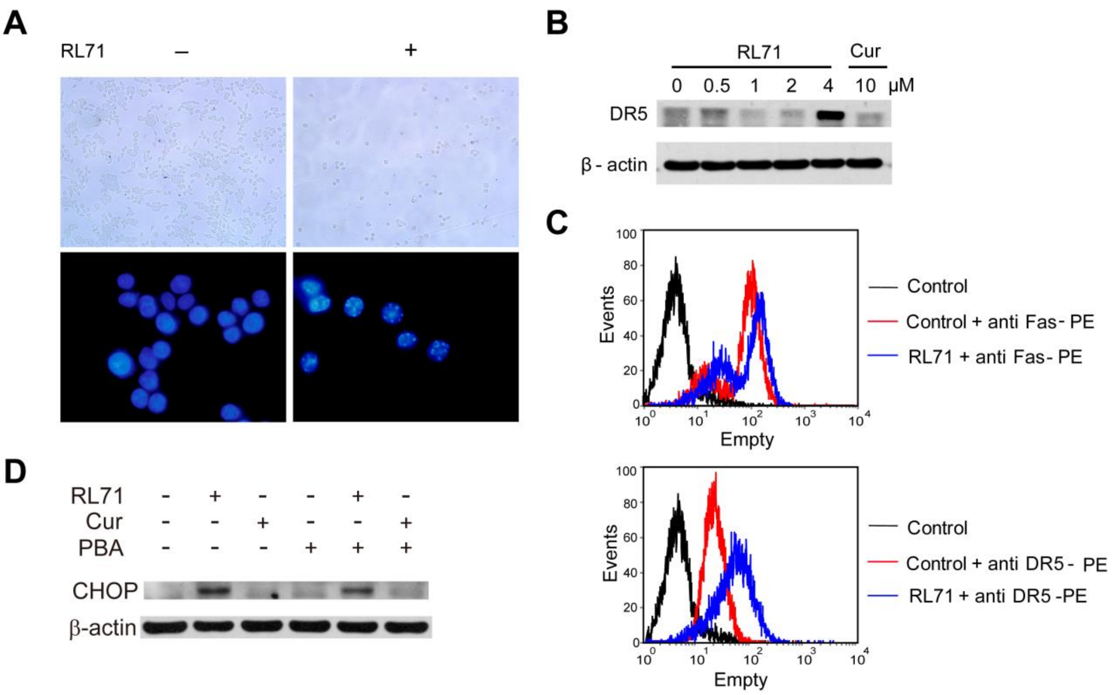

Figure S3 RL71 induces ER stress-mediated apoptosis in SW480 cells. (A) The apoptotic morphologic changes and DAPI stained karyon were photographed at 24 h after incubation with 2 μM RL71. (B) The protein levels of DR5. Cells were treated with the indicated concentrations of RL71 or Cur for 24 h. The protein levels were measured by western blot. (C) The expression of DR5 and Fas was determined by flow cytometric analysis at 24 h after incubation with 2 μM RL71. (D) The effect of PBA, a chemical chaperone, on CHOP expression. The cells were treated with RL71 (2 μM) or curcumin (10μM) with or without PBA (2.5 mM) for 24 h. The protein levels were measured by western blot. All of the flow cytometry data and western blot analyses are representative of at least 3 experiments.

Supplementary Figure S4

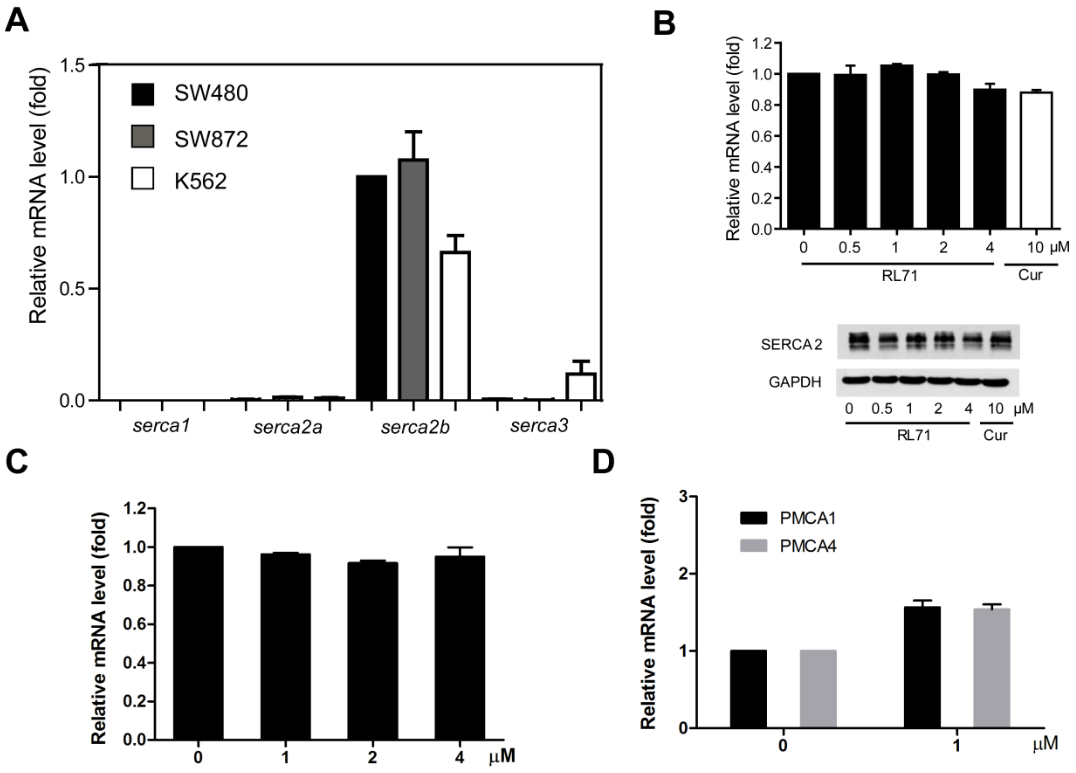

Figure S4 RL71 inhibits SERCA2 activity. (A) The relative mRNA levels of SERCA1, SERCA2a, SERCA2b, SERCA3 in SW480, SW872 and K562 cells were measured by real-time PCR. GAPDH was used as an invariant control. (B) The mRNA and protein levels of SERCA2b in SW480 cells following the treatment of indicated concentrations of RL71 or Cur for 24 h. (C) The mRNA levels of SERCA3 in K562 cells following the treatment of indicated concentrations of RL71 for 24 h. (D) The mRNA levels of PMCA1 and PMCA2 in SW480 cells treated with or without RL71.

Supplementary Figure S5

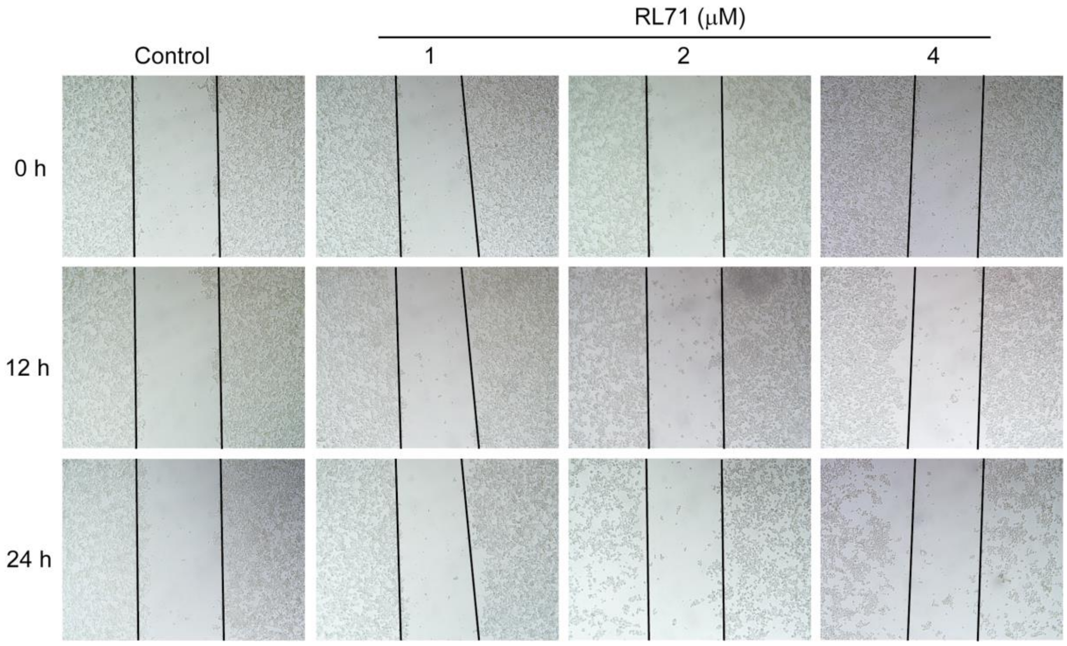

Figure S5 RL71 inhibits the migration of SW480 cells. Wound-healing assay shows that RL71 inhibited SW480 cell migration in a dose-dependent manner.

Supplementary Figure S6

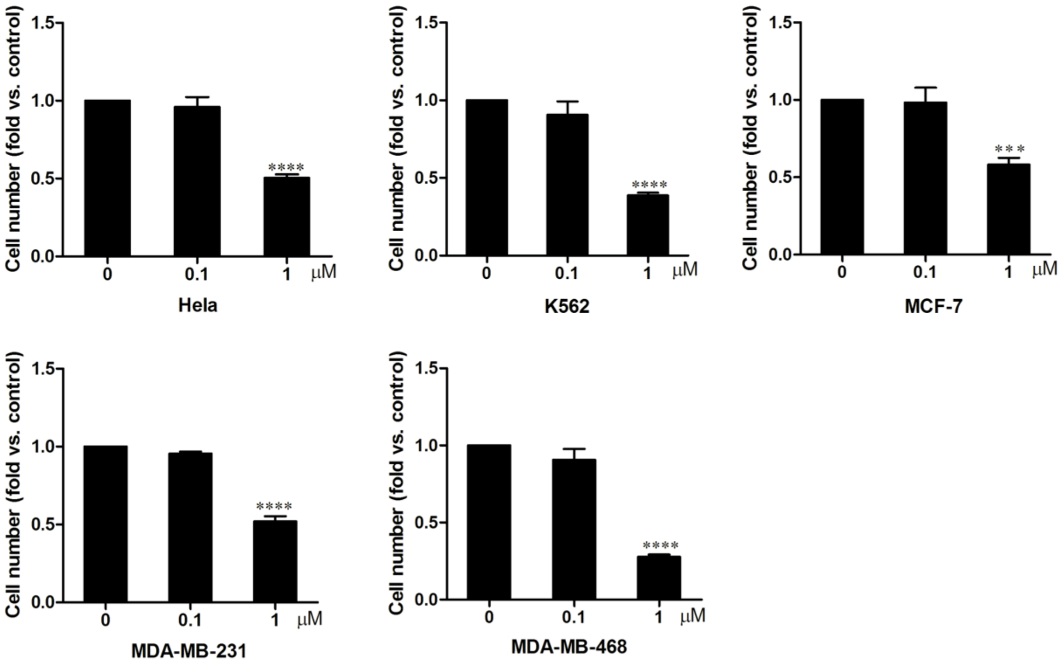

Figure S6 RL71 inhibits cell viability in other cancer cells. Cell viability was determined by MTT assay after a 48 h treatment.

Table S1. **Chemical structures and cytotoxicity of curcumin analogs in SW480 cells**

Compounds	Structure	Effect on cell viability [48h] IC_{50} ± SEM (μM)
1 (RL1)	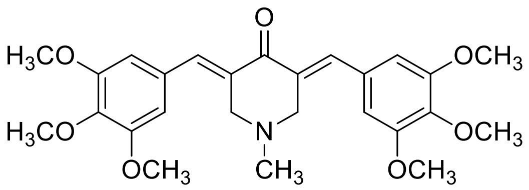	0.8 ± 0.2
2 (Curcumin)	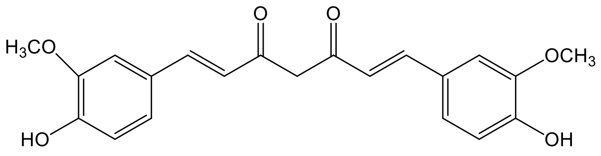	>20
3 (RL100)	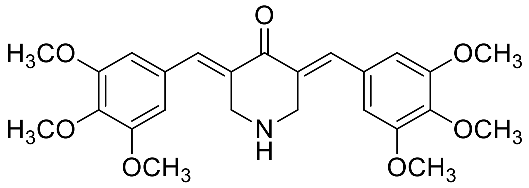	13.2 ± 1.9
4 (F36)	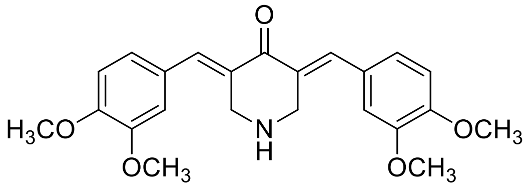	6.3 ± 1.1
5 (LH60)	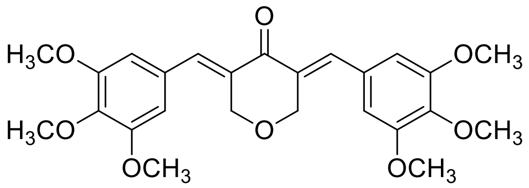	10.4 ± 2.1
6 (LH40)	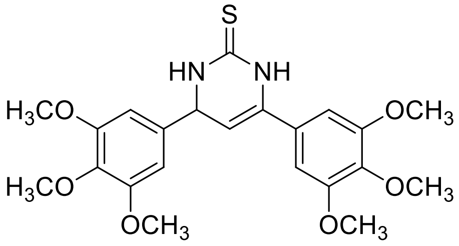	1.5 ± 0.6

7	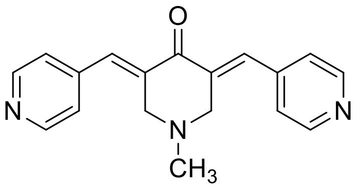	2.4 ± 0.5
8	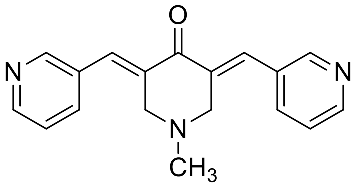	6.5 ± 0.9
9	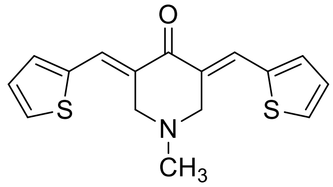	>20
10	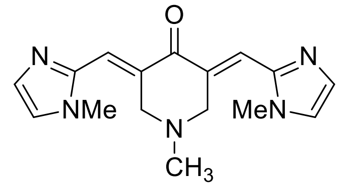	>20
11	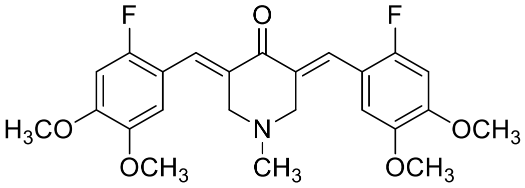	10.1 ± 1.2
12	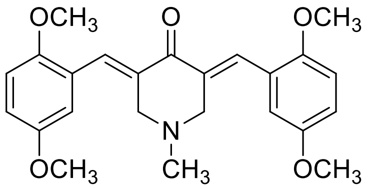	5.0 ± 0.5
13	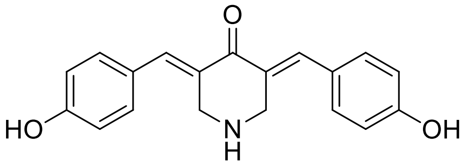	15.1 ± 5
14	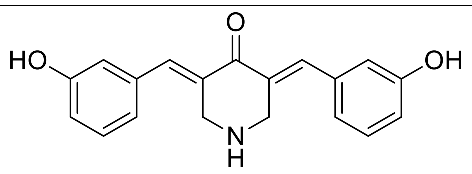	16.2 ± 3.8

Supplementary experiment procedures

Cell Lines and Culture

Human liposarcoma cell line SW872 was purchased from the American Type Culture Collection. Human chronic myelogenous leukemia K562 cells were purchased from the Shanghai Institute of Cell Biology. The K562 and SW872 cell lines were maintained in Iscove's Modified Dulbecco's Medium and DMEM, respectively, supplemented with 10% fetal bovine serum, 100 U/mL penicillin, and 100 mg/mL streptomycin and incubated at 37 °C in a humidified atmosphere containing 5% CO_2.

Wound-healing assay

SW480 cells in medium containing 10% FBS were seeded into wells of 24-multiwell plates. After the cells grew to confluence, wounds were made by sterile pipette tips. Cells were washed with PBS and refreshed with medium with 10% FBS. After incubation for 12 or 24 h at 37°C, the cells were fixed and photographed.

Real-time PCR

Quantitative PCR was performed with the ABI Prism 7000 sequence detection system (Applied Biosystems, Foster City, CA) using SYBR Green I dye (Biotium, Inc.). Conditions for amplification was one cycle of 94°C for 5 minutes followed by 35 cycles of 94°C for 30 s, 59°C for 35 s, and 72°C for 45 s. The primer sequences were shown as followed.

Primers used for amplification:

SERCA1	5'-GTGATCCGCCAGCTAATG-3'<br><br>5'-CGAATGTCAGGTCCGTCT-3'
SERCA2a	50- CTGTCCATGTCACTCCACTTCC-3'<br><br>50- AGCGGTTACTCCAGTATTGCAG-3'
SERCA2b	5'-CGCTACCTCATCTCGTCCA-3'<br><br>5'-TCGGGTATGGGGATTCAA-3'
SERCA3	5'-GATGGAGTGAACGACGCA-3'<br><br>5'-CCAGGTATCGGAAGAAGAG-3'
PMCA1	5'-CAGCAGGAGAACCAGAACCA-3'<br><br>5'-ATTCCAGCCCTCTG CACTT-3'
PMCA4	5'-TCAGGAATCCCAACGGTG-3'<br><br>5'-TCGATGACAGTGCGTACC-3'
GAPDH	5′-GGCATTGCTCTCAATGACAA-3′<br><br>5′-AGGGCCTCTCTCTTGCTCTC-3′
